# JC virus detection and JC virus-specific immunity in natalizumab-treated Multiple Sclerosis patients

**DOI:** 10.1186/1479-5876-10-248

**Published:** 2012-12-11

**Authors:** Roberta Mancuso, Marina Saresella, Ambra Hernis, Ivana Marventano, Cristian Ricci, Simone Agostini, Marco Rovaris, Domenico Caputo, Mario Clerici

**Affiliations:** 1 Don C. Gnocchi Foundation, ONLUS, Milan, Italy; 2 Department of Physiopathology and Transplantation, University of Milan, Milan, Italy

**Keywords:** Cell mediated immunity, Natalizumab, MS

## Abstract

**Background:**

The use of natalizumab in multiple sclerosis (MS) may favour JC virus reactivation; this phenomenon is usually asymptomatic but can, albeit rarely, evolve into frank progressive multifocal leucoencephalopathy (PML).

**Methods:**

JCV-specific CD8+ T lymphocytes were evaluated by flow cytometry over a 24-month period in 24 natalizumab-treated MS patients in whom JCV DNA was or was not detected in blood using quantitative real-time polymerase chain reaction; all these cases were asymptomatic.

**Results:**

Perforin- and grazymes-containing VP-1-specific CD8+ T lymphocytes were reduced whereas CD107a-expressing cells were increased in JCV positive patients, suggesting an active degranulation of these cells; naïve CD8+ T lymphocytes were also decreased whereas memory cells were increased in patients in whom JCV reactivation was observed.

**Conclusion:**

The presence of a CD8+ T lymphocyte-mediated effector immune response offers a greater insight into reactivation of JCV and its clinical sequelae, and may help the monitoring of patients on natalizumab therapy.

## Background

Natalizumab is a monoclonal antibody that binds the alpha-chain of integrins and is used in the therapy of Multiple Sclerosis (MS). Results of longitudinal studies confirmed the efficacy of such treatment, as the annual relapse rate evaluated after 1 year was reduced by 68% whereas the disability progression over 2 years was diminished by 42% in natalizumab–treated compared to placebo patients [1]. These results notwithstanding, a worrisome possible adverse effect of natalizumab is the development of progressive multifocal leucoencephalopathy (PML); recent results reported more than 200 cases of PML in natalizumab patients, with an overall incidence (1.01/1000 patients) with the greatest increase in risk occurring after 2 years of therapy [2]. PML is a severe demyelinating disease due to the lytic replication of human polyomavirus JC virus (JCV) in oligodendrocytes. PML occurs when immunosurveillance within the central nervous system (CNS) is impaired; no effective therapy is known for this condition. Risk factors for natalizumab-associated PML are still unknown. One hypothesis suggests that natalizumab could block lymphocyte trafficking through the blood–brain barrier, this would results in a decrease of immunosurveillance allowing JC virus reactivation from latency. Notably, this effect would be specific for JC virus as other opportunistic infections are only occasionally reported in natalizumab-treated MS patients [3]. Natalizumab seems also to influence bone marrow physiology. Thus, possibly as a consequence of a blocking effect of this drug on the α4β1/VCAM-1 interaction -a process pivotal in favoring the generation of T cells and B cells from bone marrow (BM) progenitors [4]- a rapid egress of CD34+ cells from the BM was observed [5]. This would create an environment favoring the reactivation of JC virus within the bone marrow [6,7].

To date, there are no methods that can reliably predict which patients have a higher risk of developing PML as no clear-cut associations can be established between JCV DNA in the blood or urine or JCV viremia and viruria and PML [8-11]. In the attempt to bypass this problem, a new assay (Viral-like particles – ELISA) was suggested to be a sensitive tool to identify patients at a reduced risk of PML development [12]; complementary tests of JCV DNA in urine were also indicated as being useful for the stratification of risk of PML in patients [13], as the detection of urinary viral DNA identifies JCV infected subject when antibody are still undetectable.

Whereas JCV viruria is frequently detected in healthy individuals, the appearance of JCV DNA in blood of such individuals is very rare. JCV-specific antibodies are present in about 58% of healthy individuals [14], but JCV reactivation and the development of PML are very seldomly observed in healthy subjects, possibly because the virus is kept at bay by cell mediated immunity. Supporting this idea are the observations that: 1) detection of JCV-specific cytotoxic T lymphocytes (CTL) in the peripheral blood of individuals with PML was shown to be a positive prognostic marker [15]; and 2) JCV-specific CTL were suggested to protect against the development of PML in JCV-infected immunocompetent individuals [16].

The mechanism underlying JC viral replication are nevertheless still unknown, and the influence of natalizumab on JCV control is unclear. We monitored the asymptomatic reactivation of JCV in peripheral blood of JCV infected MS patients treated with natalizumab over a 24-month period. Changes in cell mediated immunity parameters were compared between patients with or without JCV reactivation.

## Methods

### Study population

Twenty-four outpatients with a diagnosis of MS [17] (4 males, 20 females) were enrolled in the study at the Multiple Sclerosis Center of Don Gnocchi Foundation (Scientific Institute S. Maria Nascente, Milan, Italy) between November 2007 and June 2011. Patient’s mean age (± standard deviation) was 37 ± 10 years; mean disease duration was 12 ± 8 years with a mean Expanded Disability Status Scale (EDSS) at enrollment of 4.0 ± 1.4.

One of the patients had never been treated with disease-modifying drugs. Among the other 23 patients, 15 (65%) previously underwent immunomodulating therapies (interferon, glatiramer acetate); the other 8 individuals (35%) were treated with immunosuppressive drugs (mitoxantrone, azathyoprine). No significative differences were observed concerning the type of previous therapy, disease duration, or wash-out period prior to natalizumab in patients in whom JCV reactivation was or was not observed during therapy. All patients fulfilled the Italian Agency of Drug (AIFA) criteria for natalizumab treatment i.e. they either were affected by a particularly severe disease course in the year prior to therapy (marked clinical worsening; high relapse rate; rapid disability accumulation), or they showed a lack of response to previous immunosuppressive or immunomodulatory therapies.The washout period before initiating natalizumab was 3 months for patients previously using immune-modifying drugs, and 6 months for patients undergoing immunosuppressive therapy (mandatory according to AIFA guidelines).

Urine, blood and serum samples collected at baseline and after 1 (Mo. 1), 6 (Mo. 6), and 12 (Mo. 12) and 24 (Mo. 24) months of therapy, at the time of infusion, were utilized for JCV quantitative detection. An additional series of samples was collected within one month in the case of the detection of JC virus DNA positivity during treatment with monthly natalizumab infusions for 24 consecutive months. Finally, although not mandatory according to the AIFA guidelines, cerebrospinal fluid (CSF) analysis for JCV DNA were performed before the initiation of therapy as well as after 24 months of treatment (number of infusion: median 24, range 24–28). Written, informed consent was obtained from each patient; the study was approved by the local ethics committee.

**Figure 1:**
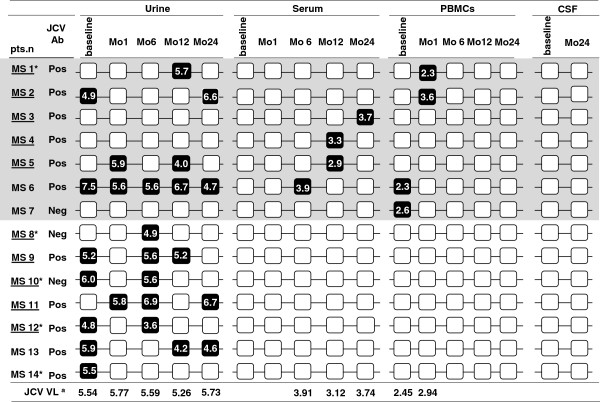
**Longitudinal testing of samples from Multiple Sclerosis patients positive for JCV DNA in urine, and/or serum, and/or PBMCs.** Black boxes correspond to positive samples, white boxes to negative samples. JCV viral load (VL) is indicated for each positive sample and median value are shown at the bottom. Patients with PBMCs analyzed for JCV-specific immunity are underlined. For each patient anti-JCV antibody status and any clinical relapse (*) during follow up are also indicated.

JCV-specific cellular immunity was prospectively evaluated using frozen peripheral blood mononuclear cells (PBMCs) specimens in 5 patients in whom JCV DNA was detected in blood (nr. 1–5 in Figure 1) and in 5 subjects in whom JCV DNA was never detected during follow-up (acting as controls) (nr. 8–12 in Figure 1). Immunological analyses were performed at baseline and after 1 (Mo. 1), 6 (Mo. 6), 12 (Mo. 12) and 24 (Mo 24) months of therapy.

### Blood sample collection

Whole blood was collected by venepuncture in Vacutainer tubes containing ethylenediamine tetra-acetic acid (EDTA) (Becton Dickinson & Co., Rutherford, NJ). PBMCs were separated on lymphocyte separation medium (Organon Teknika Corp., Durham, NC) and washed twice in phosphate-buffered saline (PBS); the number of viable leukocytes was determined by trypan blue exclusion.

### Detection of JCV antibodies

After the activation of a centralized service supported by a grant from Biogen Idec (STRATIFY project), enzyme linked assay (ELISA) was performed to detect antibodies specific for JCV virus-like particles [12] in serum of 23/24 enrolled subjects treated for a 24-month period.

### Detection of viral genome by qPCR

DNA was extracted from 150 μl of cell-free CSF, serum, or urine using the spin-columns technique, or from 5x10^6^ PBMCs (Nucleospin virus kit and Nucleospin Tissue kit, Macherey-Nagel, Duren, Germany) according to the manufacturer instructions. Detection and quantification of JCV DNA was performed by qPCR targeting the large T-antigen gene of JCV genome, following a previously described protocol [18]. Limit of detection (LOD) was 2 copies/μl of extracted DNA (corresponding to 10 copies/reaction or 500 copies/ml of CSF or serum or urine, or 40 copies/μg of DNA from PBMCs). All samples were assayed in triplicate, leading to 3 fold increasing of analytical sensitivities (166 copies/ml or 2.3 Log_10_; 13 copies/μg DNA or 1.1 Log_10_). Samples resulting positive in only one replicate were re-extracted and retested in triplicate.

All laboratory procedures were carried out under stringent conditions to avoid contamination. In details, samples were processed individually and DNA extractions were performed one at time, in a clean, dust-free area with sterile and disposable materials, with a change of gloves for each sample.

### Selection and synthesis of VP-1peptides binding to MHC molecules

The VP-1 JCV protein was chosen since this protein has been shown to be preferentially recognized by CD8+ T lymphocytes [19]. Binding properties of VP-1 peptides to Class I molecules were predicted using the RANKPEP program (Molecular Immunology Foundation at the site http://www.mifoundation.org/Tools/rankpep.html).

Fourteen 9 mer with high score and two 9 mer VP-1 peptides with the lowest score were selected, as antigenic and negative controls respectively, to evaluate HLA-I-restricted JCV specific responses. Peptides were synthesized using Fmoc chemistry; their purity, as assayed by HPLC, was >70% and their composition was verified by mass spectrometry [20]. Lyophilized peptides were dissolved at 25mg/ml in DMSO or sterile water to prepare peptide pools (10 μg/ml final concentration).

All the peptides have been previously used to asses JCV- specific immune responses by proliferation assay and intracellular cytokine production. Preliminary experiments (data not shown) were performed with PBMC of 5 PML patients and 5 healthy controls (HC); results confirmed previously published data [21,22].

CFSE proliferation assay and intracellular cytokine detection in unstimulated PBMC or in PBMC that were stimulated with the VP-1 peptide pools or the peptide control pool confirmed that proliferation of IFN-γ or TNF- α-producing CD8+ T cells could be detected upon stimulation with the antigenic VP-1 peptides alone.

### VP-1-specific CD8+ T cell subsets

1x10^6^ PBMCs were placed in polystyrene tissue culture plates containing 1 ml RPMI +10% AB human serum, 10 μl of CD107a [23], and either non-immunogenic (unstimulated) or VP-1 peptides. Plates were incubated at 37°C in a humidified 5% CO2 atmosphere for 5 hours. Brefeldin A (Sigma) (final concentration 10 μg/ml), was added after 1 hour of incubation. Cells were then harvested, washed with phosphate-buffered saline (PBS), stained with an anti-CD8 mAb, and incubated 30 min at RT in the dark. Cells were subsequently washed with PBS and 100 μl of Saponin 0.5% was added to permeabilize the cell surface. Ten μl of IFN-γ, TNF-α, perforin- and granzyme-specific mAb were then added to the cells that were finally washed with PBS and resuspended in 500 μl of PFA 1%.

### VP-1 specific-CD8+ T cell Subsets

1x10^6^ PBMCs were placed in polystyrene tissue culture plates containing 1ml RPMI +10% AB human serum, and either non-immunogenic (unstimulated) or VP-1 peptides for 5 hours at 37°C in a humidified 5% CO2 atmosphere. Cells were then harvested, washed with PBS, stained with an anti-CD8 anti-CD45RA and anti-CCR7 mAb, and incubated 30 min at RT in the dark. Finally stained cells were fixed in 500 μl of PFA 1%. The following CD8+ Tcell subsets were analyzed by flow-cytometry: naïve (CD8+45RA+CCR7+), central memory (CM) (CD8+45RA-CCR7+), effector memory (EM) (CD8+45RA-CCR7-) and terminally differentiated (TD) (CD8+45RA+CCR7-).

### Monoclonal antibodies (mAbs)

The following mAbs were used: anti-Phycoerythrin-Cyanin-7 (PC7)-labeled anti-CD4 (clone SFCI12T4D11), Phycoerythrin-Cyanin-5 (PC5)-labeled anti-CD8 (clone B9.11), Phycoerythrin (PE)-labeled anti-CD3 (UCHT1) (IgG1), FITC-labeled anti-TNFα- (MP9-20A4), PE-labeled anti-IFNγ- (B27)(both m IgG1) (Invitrogen-Caltag Lab Carlsbad, CA, USA), FITC-conjugated anti**-**perforin (δG9) (m IgG2b), and PE conjugated anti**-**granzyme- (GB11) (m IgG1) (Becton**-**Dickinson Biosciences, San Jose CA, USA) anti-human CD107a PE-labeled (clone H4a3, isotype mouse IgG 1k BD Biosciences, San Jose, CA) as well as isotype-matched mouse mAbs were also utilized.

### Statistical analysis

Statistical analysis of prevalence data at each time point in urine, serum and blood samples were carried out with two-sided Fisher’s exact test. Quantitative continuous variables (JCV viral load) were not normally distributed and comparisons were performed using non parametric test. A repeated measures analysis of variance was used to investigate for variable change (perforin, granzyme, CD107a) after virus activation. In this model measures following virus activation were marked as dummy and within-subject analysis was performed. The P value on the graph was performed by Fisher’s method for independent measures and was calculated considering P values from Mann–Whitney U-test comparing subject with virus activation to subject without virus activation at any observation across time. Independent measures hypothesis was tested by means of Hoeffding test. All statistical evaluation was performed by SAS software vers.9.2.

## Results

### Clinical outcome

A clearly positive outcome of the study was that no PML cases developed during the follow-up period. Clinical relapses during the 24-month period of therapy were observed in 7/24 patients; anti-natalizumab antibodies were detected in one of these cases. All these patients were successfully treated with IV methylprednisolone (IVMP) for 3 days and natalizumab treatment was continued. Data of 5 of the patients in whom a relapse during follow up was observed are presented in Figure 1 (nr. MS1, MS8, MS10, MS12,MS14*)*; DNA could never be isolated from the other 2 individuals and, as a consequence, data of these 2 patients are not shown in this figure.

Twenty-four patients, reached a follow-up of 24 months. The clinical efficacy of Natalizumab was confirmed by the observation that the EDSS remain stable, or was reduced in 23/24 patients (96%). Mean EDSS score showed a reduction from baseline (4.0 ± 1.4) to post–treatment (3.8 ± 1.6) even if it the changes were not statistically significant (p=0.61).

### JCV in urine, serum, PBMCs and CSF

JCV was detected at baseline and/or during follow-up in urine, serum and/or PBMCs in 14/24 (58%) patients. Figure 1 shows virological data obtained from these 14 patients; JCV DNA was never observed in the remaining 10 individuals.

JCV was observed in urine in 11/14 cases, in serum in 4/14 individuals, and in PBMCs in 4/14 cases. JCV was detected in multiple sites (e.g. urine and serum) in 4/14 patients whereas it was present in a single specimen in the other 10 cases (see Figure 1). No differences were observed between the JCV-positive and the JCV-negative patients either in age or gender (data not shown). All the CSF samples tested were JCV negative both at baseline and after 24 months of treatment (Figure 1).

### Anti–JCV antibodies

12/24 (50%) individuals resulted seropositive for JCV. Only in 3 of these cases seropositivity did not coincide with JCV detectability by qPCR. In 3 other cases the opposite situation was observed: thus, these 3 patients were JCV seronegative but JCV DNA could be detected by qPCR (two in urine, 1in PBMC; MS7, MS8, MS10 in Figure 1).

### Longitudinal assessment of JCV in MS patients

#### JCV in urine

Consistent with other studies [24,25] the overall frequency of JCV viruria was 28% at baseline, and respectively 12%, 24%, 20% and 16% after 1, 6, 12, and 24, months of natalizumab treatment. JCV viruria was detected in at least one sample during follow-up in 11/24 (42%) cases (Table 1); no significant differences were observed in JCV viral load in urine (Log_10_ copies/ml) when baseline results (median: 5.54, IQR: 4.96-5.97) were compared to data obtained at the different time of follow-up (Mo1: 5.77, 5.66-5.90; Mo6: 5.59, 4.94-5.62; Mo12: 5.26, 4.15-5.94; Mo24: 5.73, 4.78-6.67).

**Table 1:** JCV detection in urine, serum and PBMCs of natalizumab-treated MS patients

	**Patients (nr)**	**Median VL^a^**	**Median VL (IQR)^b^**
PBMCs	4/24*	3 x 10^2^	2.45 (2.28-3.11)
serum	4/24^#**†**^	3.75 x 10^3^	3.53 (3.11-3.82)
urine	11/24*^†^	4.1 x 10^5^	5.60 (5.08-5.84)

^a^ Viral loads (VL) are expressed as viral copies/ml in urine and in serum, as copies/μg of DNA in PBMCs. ^b^VL median values and interquartile range (IQR) are expressed as Log_10_ copies.

* ^**†**^ Fisher exact test : PBMCs or serum vs. urine p= 0.062.

Longitudinal analysis of virus detection in JCV positive patients is shown in Figure 1; JCV viruria was observed frequently at a single time point, but only in one case (patient 6) a persistent JCV viruria during the follow-up was detected; notably this was the only individuals in whom JCV was detected in all three investigated sites (urine, serum, PBMCs), without any evidence of PML.

#### JCV in blood

JCV was detected in peripheral blood in 7 patients (two cases in PBMC at baseline; five cases in serum/PBMC during treatment): in all these patients JCV was negative in subsequent samples (within one month after the positivity). Median JCV DNA viral load in PBMC was 2.45 Log_10_ copies/μg DNA; JCV DNA median viral load in serum was 3.53 Log_10_ copies/ml (Figure 1).

### Cytokine-producing CD8+ T lymphocytes

JCV-specific cell mediated immune parameters were evaluated in 5 patients in whom JCV DNA was detected in peripheral blood (JCV POS: patients nr. 1–5 in Figure 1) during treatment, and in 5 subjects in whom JCV was never detected during follow-up in peripheral blood (JCV NEG: patients nr. 8–12 in Figure 1).

**Figure 2:**
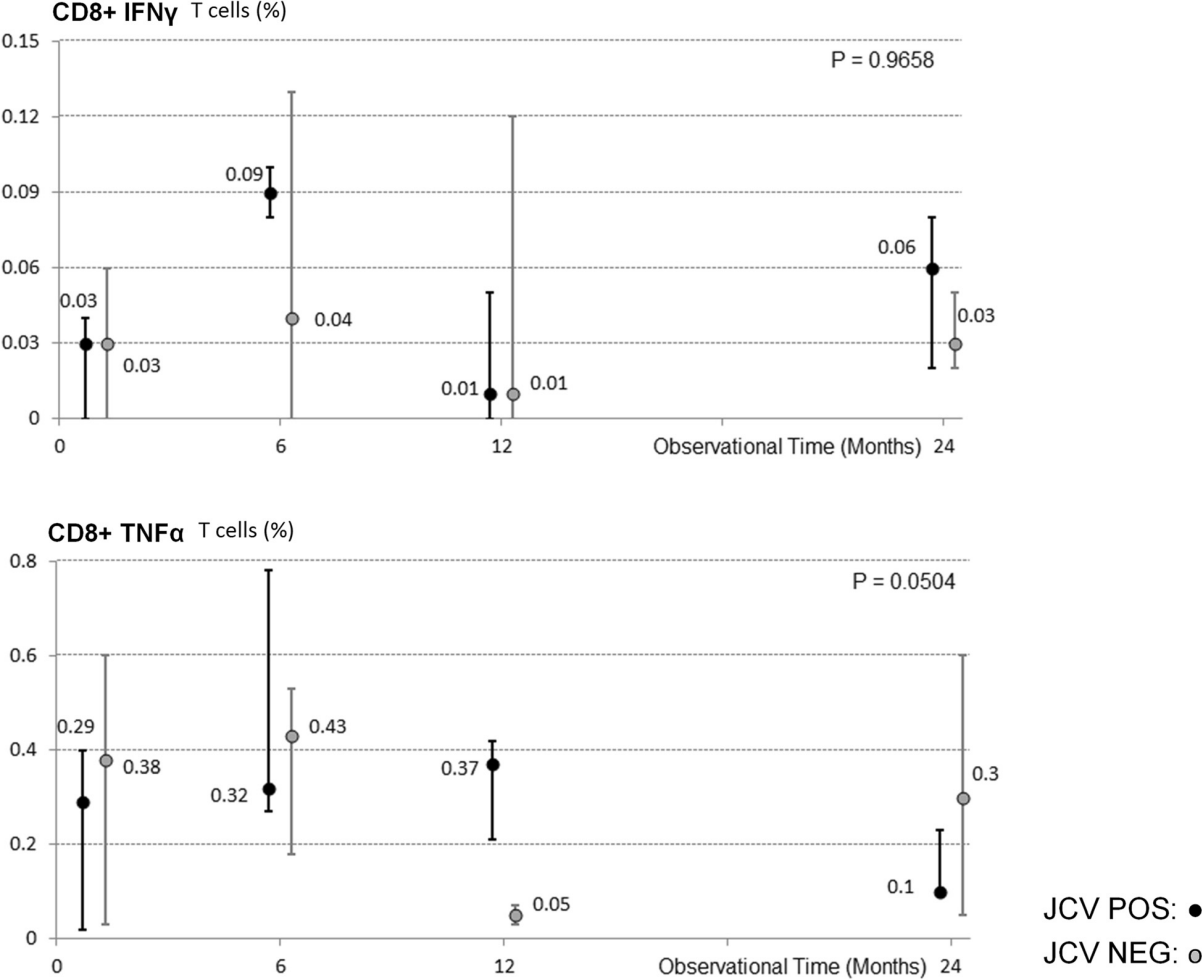
**VP-1 stimulated IFN-γ and TNF-α cytokine-producing CD8+ T lymphocytes in Multiple Sclerosis patients undergoing natalizumab.** Results obtained over a 24 months period are shown; grey dots indicate subject in whom virus activation in blood was not detected (JCV NEG); black dots indicate individuals in whom JCV DNA was detected in blood (JCV POS). Error bars depict first and third quartiles and median values are indicated. Statistical significance is presented.

VP-1-stimulated tumour necrosis factor-alpha (TNF-α)- and interferon-gamma (IFN-γ) - producing CD8+ T lymphocytes were measured to determine possible modifications in the frequency of cytokine-producing circulating VP-1-stimulated T cells. A repeated measures analysis of variance was used to investigate for variable change in patients with virus reactivation in blood. In this model, measures following virus activation were marked as dummy and within-subject analysis was performed. Intra-subjects analysis of results did not show the presence of significant variations in any of the cytokines analyzed in two groups analyzed (Figure 2).

### VP-1-stimulated perforin- and granzymes- containing CD8+ T lymphocytes

**Figure 3:**
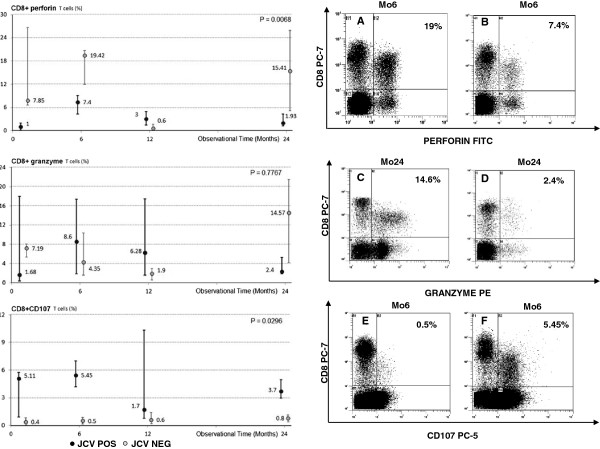
**VP-1 stimulated perforin-, granzyme-, or CD107a-expressing- CD8+ T lymphocytes in Multiple Sclerosis patients undergoing natalizumab.** Left Column: Results obtained during a 24 months period; grey dots indicate subject in whom virus activation in blood was not detected (JCV NEG); black dots indicate individuals in whom JCV DNA was detected in blood (JCV POS). Error bars depict first and third quartiles. Statistical significance is shown. Right Column: Representative results obtained in JCV NEG (**A**, **C**, **E**) or in JCV POS (**B**, **D**, **F**) patients. In the upper right corner the percentage of positive cells is indicated.

Perforin and granzymes mediate the destruction of virus-infected cells upon being released by antigen-specific CD8+ T lymphocytes; VP-1-stimulated granzymes and perforin containing CD8+ T lymphocytes were analyzed in both groups of patients. Results showed that VP1-stimulated perforin- and granzyme-containing CD8+ T cells were differently expressed in JCV POS compared to JCV NEG patients (Figure 3); notably the levels of both proteins was reduced in JCV POS individuals. The differences reached statistical significance in the case of perforin.

### VP-1-stimulated CD107a-expressing CD8+ T lymphocytes

CD107a is expressed on the surface of CD8+ cytotoxic T lymphocytes upon degranulation. CD107a-expressing VP-1-specific CD8+ T cells were analyzed in all patients; a repeated measures analysis of variance was used to investigate for variable change in patients with virus reactivation in blood. Results indicate that these cells were significantly augmented in JCV POS patients, suggesting that CD8+ T cells do indeed degranulate in response to viral DNA (Figure 3).

### VP-1-specific CD8 T cell subsets

**Figure 4:**
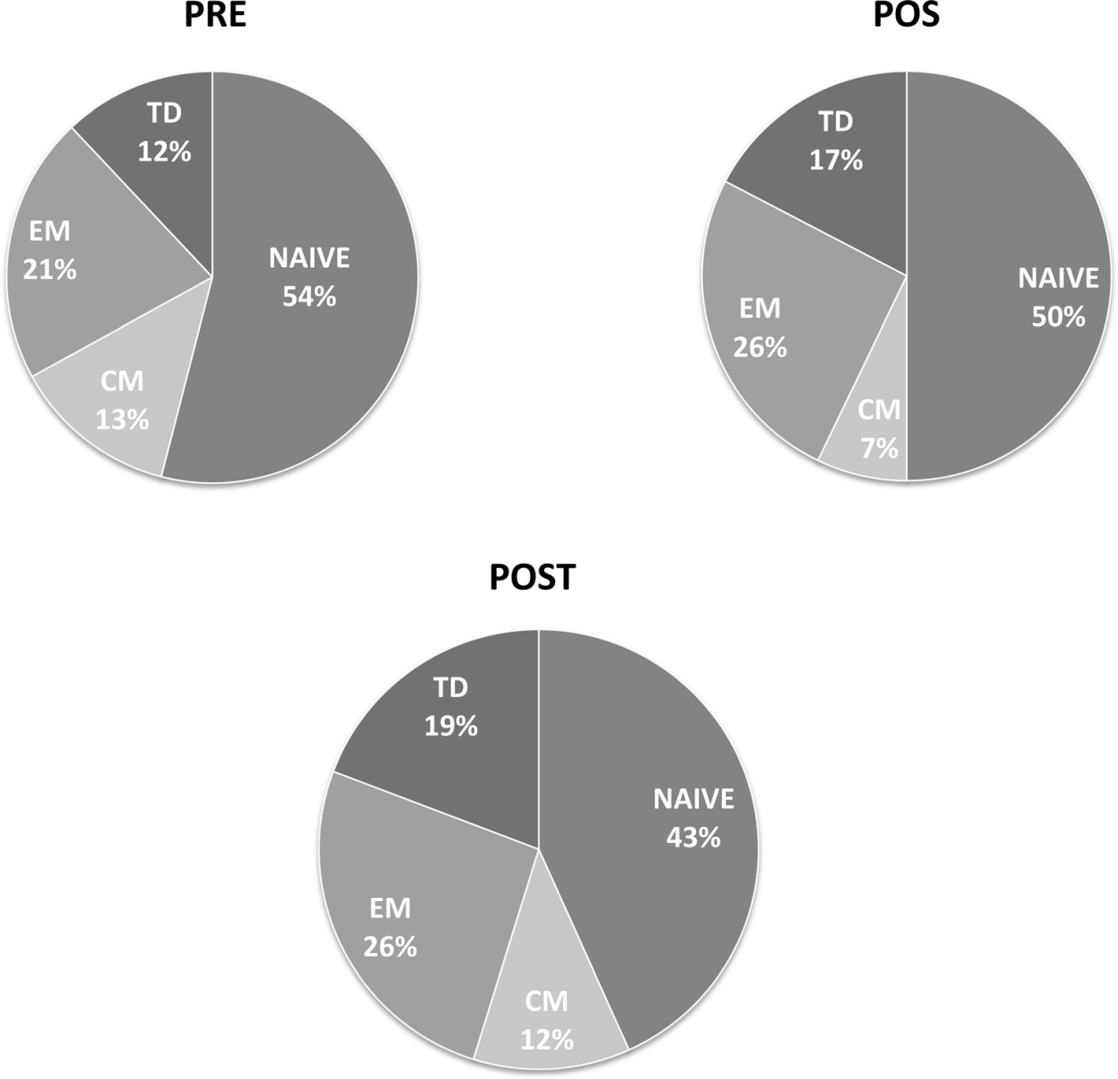
**Memory/Naïve T lymphocyte subsets.** Relative proportions of VP-1 stimulated CD8+naïve (CCR7+CD45RA+), central memory (CM: CCR7+CD45RA-), effector memory (EM: CCR7-CD45RA-), and terminally differentiated (TD: CCR7-CD45RA+) T lymphocytes in Multiple Sclerosis patients undergoing natalizumab. Samples were tested at baseline (PRE), at the time of JCV DNA isolation in blood (POS), and after 24 months of therapy (POST).

Subsetes of VP-1-specific naïve and memory CD8+ T lymphocytes were examined next. The following lymphocytes subsets were analyzed: naïve (CD8+/CDRA+/CCR7+), CM (Central Memory: CD8+/CDRA-/CCR7+), EM (Effector Memory: CD8+/CDRA-/CCR7-) and TD (Terminally Differentiated: CD8+/CDRA+/CCR7-) cells. Results showed that VP-1-specific naïve (CD8+/RA+/CCR7+) cells were diminished (PRE: median 54%, IQR48-54; POS 50%, IQR 48–56; POST: 43%, IQR 18–50) whereas effector memory (CD8+/RA-/CCR7-) (PRE: 21%, IQR 16–33; POS: 26%, IQR 24–31; POST: 26%, IQR 26–53) as well as terminally differentiated (CD8+/RA+/CCR7-) (PRE: 12%, IQR 7–13; POS: 17%, IQR 10–14.5; POST 19%, IQR 12–17) lymphocytes were increased in JCV POS individuals (Figure 4). Because differentiation of naïve T lymphocytes into memory populations is contingent upon antigen recognitions, these results indeed suggest that JCV replication took place in JCV POS patients during follow-up.

## Discussion

JC virus, the cause of PML, a demyelinating disease of the central nervous system observed in immune-compromised individuals, is one of the most prevalent viruses in humans. Thus, urinary excretion is common in healthy Europeans [26,27], and JCV DNA viruria is detectable in about one half of healthy peoples living in Italy [28]. Notably, long-term or continuous JCV urinary excretion has been observed in 20 to 50% of healthy individuals [27], with a wide variation of the quantities of JCV excreted in urine within different subjects [29].

It recently became clear that the use of natalizumab, a monoclonal antibody used in the therapy of MS, can result in the reactivation of JCV in CNS [30]. The connection between natalizumab and JCV reactivation is not known, even if it appears clear that alterations in immunosurveillance within the CNS are involved in this phenomenon. The effect of natalizumab on JCV in MS patients has been analysed, with conflicting results: some authors [31,32] reported the frequent detection of asymptomatic JCV reactivation in the urinary tract after therapy; these observations were nevertheless not replicated in other analyses [24,25].

It is debated whether the detection of JCV in blood and/or plasma results in a higher likelihood to develop PML. Thus, JCV DNA can sometimes be detected in blood in healthy individuals [33,34], but viral load in blood is usually very low even in patients with a diagnosis of PML [35]. The connection between JCV and PML is nevertheless reinforced by the observations that: 1) the estimated hypothetical PML incidence in JCV Ab-negative patients is extremely low (0.09 cases per 1000 patients) [2]; and 2) no PML cases have ever been reported in JCV Ab-negative individuals [36].

Humoral immunity alone cannot control JCV replication [37-39] as JCV shedding in urine of immunocompetent individuals is very common, suggesting that also cellular immunity seems to be not very efficient in JCV replication control [14].

To verify whether correlations could be observed between the appearance of JCV DNA in blood and the elicitation of virus-specific cell mediated immunity we analyzed this arm of the immune response in patients in whom blood viral DNA was, or was not, detected. Results herein show that a reduced quantity of VP-1-stimulated CTL containing the proteins that, upon degranulation, are responsible for the destruction of virus infected target cells via lytic (perforin) and apoptotic (granzymes) mechanisms is observed in those patients n whom JCV DNA was detected in blood. CD107a is expressed by CTL only when these cells release perforin and granzymes. Notably, VP-1-specific CD107a-expressingCD8+ T lymphocytes were increased in patients in whom JCV DNA was detected during follow-up, indicating that the diminished number of JCV-specific perforin- and granzymes-positive lymphocytes is a consequence of degranulation i.e. of an active virus-specific and CTL-mediated immune response. That virus replication indeed occurred in a subset of natalizumab-treated patients is further confirmed by the observation that a differentiation from naïve toward effector phenotypes of VP-1-specific CD8+ T lymphocytes was observed in such patients.

## Conclusions

Taken together these results indicate that the appearance of JCV DNA in blood of natalizumab treated MS patients is an event associated with the differentiation of virus-specific cells and the elicitation of a CD8+ T lymphocyte-mediated immune effector response. It is tempting to speculate that this response is responsible for the containment of JCV reactivation and for preventing the clinical consequences of such reactivation.

JCV-specific cellular immune responses were recently shown to diminish over time in natalizumab-treated MS patients in whom viremia developed [31].

Our results, even if obtained in a small group of patients and needing to be replicated and expanded, offer a mechanistic explanation justifying on an immunological basis why the appearance of JCV DNA in blood does not result in symptomatic JCV reactivation; moreover it is necessary to consider, as previously suggested [14], that JCV replication (shedding in urine) could be not necessarily a sign of T cell disfunction, but instead reflect differences in presentation and/or processing of viral antigens, anatomic location and/or accessibility to T cells.

This study was designed to compare MS patients in whom JCV did or did not reactivate during therapy; given high prevalence of JCV infection in the population at-large it will be interesting to verify JCV-specific CMI in JCV- infected healthy individuals.

A final consideration is that, based on the preliminary results herein, investigating the impact of new monoclonal therapies (e.g. efalizumab, rituximab, infliximab) on virus specific cell mediated immune response could be useful to better clarify the mechanisms associated with JCV reactivation.

## Competing interests

The authors declare that they have no competing interest.

## Authors’ contributions

RM, MC designed the experiments; MR, DC recruited patients and collected clinical data; AH,IM,SA, performed experiments and data collection ; CR, RM, MS were involved in data analysis; MC, MS, RM interpretation of the data and drafting the manuscripts. All the authors revised and approved the final manuscripts.
